# Acoustic Signal-Based Defect Identification for Directed Energy Deposition-Arc Using Wavelet Time–Frequency Diagrams

**DOI:** 10.3390/s24134397

**Published:** 2024-07-07

**Authors:** Hui Zhang, Qianru Wu, Wenlai Tang, Jiquan Yang

**Affiliations:** Jiangsu Key Laboratory of 3D Printing Equipment & Manufacturing, School of Electrical and Automation Engineering, Nanjing Normal University, Nanjing 210023, China; huizhangzh1@163.com (H.Z.); yangjiquan@njnu.edu.cn (J.Y.)

**Keywords:** defect identification, convolutional neural network, acoustic signals, wire arc additive manufacturing

## Abstract

Several advantages of directed energy deposition-arc (DED-arc) have garnered considerable research attention including high deposition rates and low costs. However, defects such as discontinuity and pores may occur during the manufacturing process. Defect identification is the key to monitoring and quality assessments of the additive manufacturing process. This study proposes a novel acoustic signal-based defect identification method for DED-arc via wavelet time–frequency diagrams. With the continuous wavelet transform, one-dimensional (1D) acoustic signals acquired in situ during manufacturing are converted into two-dimensional (2D) time–frequency diagrams to train, validate, and test the convolutional neural network (CNN) models. In this study, several CNN models were examined and compared, including AlexNet, ResNet-18, VGG-16, and MobileNetV3. The accuracy of the models was 96.35%, 97.92%, 97.01%, and 98.31%, respectively. The findings demonstrate that the energy distribution of normal and abnormal acoustic signals has significant differences in both the time and frequency domains. The proposed method is verified to identify defects effectively in the manufacturing process and advance the identification time.

## 1. Introduction

Directed energy deposition-arc (DED-arc) is a directed energy deposition technology that employs an electric arc as a heat source and metal wires as raw material, which are melted and then deposited layer-by-layer to obtain the formed parts [1,2]. Based on the type of heat source, the DED-arc process can be categorized into gas metal arc welding (GMAW), gas tungsten arc welding (GTAW), and plasma arc welding (PAW) [3,4]. In comparison with other additive manufacturing technologies, DED-arc is distinguished by its high deposition rate, high material utilization, and low cost, enabling the production of large metal parts [5,6,7]. It is therefore widely employed in the aerospace and other industrial manufacturing fields [8,9]. However, because of the instability of the DED-arc process, defects such as cracks, lack of fusion, residual stress, and pore defects may occur during the forming process, which negatively impacts the performance of the formed parts [10]. The lack of fusion is defined as the failure of the material to melt completely and fill the weld bead because of insufficient heat input [11]. This results in the formation of areas of inadequate metallurgical bonding, which may manifest as a discontinuous weld bead. The repeated heating and cooling of the material results in the generation of high residual stresses within the part, which can lead to delamination, warpage, cracks, and other defects [12]. Pore defects are the result of the intrusion of external gases or contaminants or inadequate gas stripping within the melt pool [13]. Furthermore, the manufacturing process is highly dependent on manpower with low automation and intelligence. Consequently, to guarantee the quality of the formed parts, it is of the utmost importance to develop a dependable in situ monitoring system that is capable of detecting defects that may arise during the forming process in a timely and accurate manner.

Significant progress has been made in the field of artificial intelligence (AI) in recent years, with numerous researchers demonstrating a keen interest in AI techniques for defect recognition in metal additive manufacturing. Indeed, AI algorithms have been applied in several studies on DED-arc process monitoring. Xia et al. [14] employed several convolutional neural network (CNN) models to classify images of hump, spatter, torch stop, and normal molten pools, and they found that the classification accuracy of the utilized CNN models exceeded 97%. In order to classify molten pool images as normal or abnormal, Cho et al. [15] developed a CNN-based image classification model, in which MobileNetV2 had a classification accuracy of up to 98%. To address the issue of YOLO4 failing to identify minor defects in formed parts based on images, Li et al. [16] developed an enhanced model based on YOLO4 to monitor surface pore, groove, and slag inclusion. Zhang et al. [17] constructed a series of CNN models to identify the liquid, half-solidified, and solidified zones of molten pools accurately based on the melt pool images. Additionally, AI has been employed in laser-directed energy deposition (L-DED) and laser-powder bed fusion (L-PBF) processes for the purpose of in situ monitoring. In a recent study, Yu et al. [18] proposed an innovative feature fusion deep learning method for the in situ monitoring of the L-DED process and the prediction of the weld bead geometry. This method fused molten pool images and process parameters, including laser power, scanning speed, and powder feed rate, to significantly improve prediction accuracy. Li et al. [19] employed a signal-to-image methodology to integrate diverse data types, including images, acoustic signals, and photoelectric signals, for the real-time quality monitoring of L-PBF based on a CNN model. In a study by Zhang et al. [20], the accuracy of an SVM model based on manually extracted features including the melt pool, plume, and spatter was compared with that of a CNN model based on the original melt pool image data for the classification of L-PBF quality grades. The results demonstrated that the CNN model based on the original data exhibited superior classification performance. It can be seen that most monitored data are image signals, and CNN is capable of learning complex feature representations efficiently from image data, which is crucial for enhancing the accuracy of defect identification. The utilization of cameras for the purpose of image capture is an intricate and costly process, encompassing both the initial operation and subsequent processing. The monitoring method based on acoustic signals has the potential to address the aforementioned issues. While in situ monitoring based on acoustic signals has been employed in L-PBF and L-DED, there are fewer applications in DED-arc. This study explored the potential for extending in situ monitoring based on acoustic signals to DED-arc.

Acoustic signal analysis has emerged as a promising non-destructive inspection method because of its simplicity, real-time capability, and low cost [21]. In the case of GMAW, Grad et al. [22] discovered that arc acoustic signals exhibit distinct characteristics in different welding conditions and that they contain a wealth of information that can be utilized as a basis for the identification of defects. By analyzing the time–frequency characteristics of acoustic signals, it is possible to capture the characteristic information generated by defects effectively. Zhang et al. [23] employed Fisher distance and principal component analysis to extract the frequency components of an acoustic signal associated with defects. Following this, a classification model incorporating support vector machines, grid search optimization, and cross-validation was constructed for the identification of under penetration, normal penetration, and burning through. Surovi et al. [24] extracted acoustic signal features based on Mel frequency cepstral coefficients (MFCCs) and applied machine learning models to identify geometrical defects in DED-arc with an accuracy of greater than 80%. The MFCC is primarily concerned with the frequency domain, whereas the wavelet time–frequency diagrams offer a comprehensive representation of both the time and frequency domains of signals. The wavelet transform, a powerful time–frequency analysis tool, provides a time–frequency representation of signals, thereby revealing the local characteristics of signals. Acoustic signals are one-dimensional (1D) signals, whereas standard CNN architectures require two-dimensional (2D) image data as input [25]. The utilization of one-dimensional time series signals in conjunction with two-dimensional image signals has been previously employed in the context of L-PBF process monitoring. By combining high-speed synchrotron X-ray imaging and thermal imaging, Ren et al. [26] converted the time-series signals of the average emission intensity from keyhole regions into wavelet time–frequency diagrams. These diagrams were then utilized to facilitate the real-time detection of keyhole porosity through a CNN. Drissi-Daoudi et al. [27] employed a CNN model to identify different process regimes in LPBF through the analysis of acoustic signals and time–frequency diagram extraction. It is possible to visualize the energy distribution of acoustic signals at different times and frequencies using wavelet time–frequency diagrams, providing an intuitive basis for defect identification [28]. The combination of the powerful learning capability of CNNs and the feature extraction capability of wavelet time–frequency diagrams can further enhance the performance of DED-arc defect identification.

A considerable number of studies have been conducted based on AI algorithms to monitor defects in the DED-arc process through molten pool images. Nevertheless, there is a scarcity of studies on DED-arc defect monitoring based on acoustic signals. This paper introduces an investigation into the monitoring of the DED-arc process using sound-based techniques. The objective is to develop a method for identifying DED-arc defects based on acoustic signals. The method employs the wavelet transform to convert acoustic signals into time–frequency diagrams to perform time–frequency analysis. In order to achieve the objective of defect monitoring, this study proposes the application of CNNs for the classification of time–frequency diagrams. The approach enables the identification of defects in real time with high accuracy, thereby enhancing the automation and intelligence of DED-arc.

## 2. Materials and Methods

**Figure 1:**
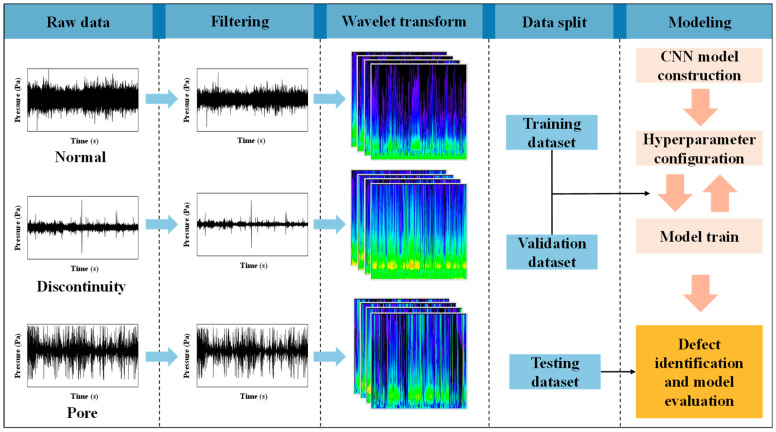
Overall workflow for defect identification.

DED-arc utilizes an arc to melt metal wires and deposit droplets in a layer-by-layer manner as the welding torch moves. Because of the inherent instability of the arc, a variety of defects may arise during the welding process, which can have a detrimental impact on the forming shape and performance of the formed parts [29]. In order to verify the initial effectiveness of the method in this study, the discontinuity and pore defects that are simpler to analyze were selected. This provided a solid foundation for the potential application of the method to other defect types. The arc acoustic signals encompass a multitude of information pertaining to the fabricating process [30]. Following the conversion of the acoustic signals into wavelet time–frequency diagrams, it became possible to consider the time and frequency characteristics of the signals in detail, thereby providing a foundation for the identification of defects. This study proposes a novel approach to train CNN models using 2D time–frequency diagrams converted from 1D acoustic signals generated during the welding process. The CNN models were used to extract features of the time–frequency diagrams, which were used to distinguish among normal conditions, discontinuity defects, and pore defects. Figure 1 provides an overview of the data processing and defect identification system.

The framework of this study is as follows. Initially, the acoustic signals of distinct welding conditions were gathered via experimentation, and wavelet denoising was used to eliminate excess noise. The denoising process employed in this work utilized the db4 wavelet with soft thresholding and four levels of decomposition. Then, the sliding window was applied to segment the data into batches of suitable size to expand the datasets. The next step in the process was time–frequency diagram conversion. The segmented acoustic signal samples were converted into wavelet time–frequency diagrams using wavelet transform to obtain and then label the dataset. The dataset was separated into distinct subsets, namely, a training set, a validation set, and a testing set, separately. The training set served as the basis for model training. The validation set was employed to fine-tune the hyperparameters of the model. The testing set was utilized for model assessment. Several classical and lightweight CNN architectures were investigated, including AlexNet, ResNet-18, VGG-16, and MobileNetV3.

### 2.1. Experimental Setup

**Figure 2:**
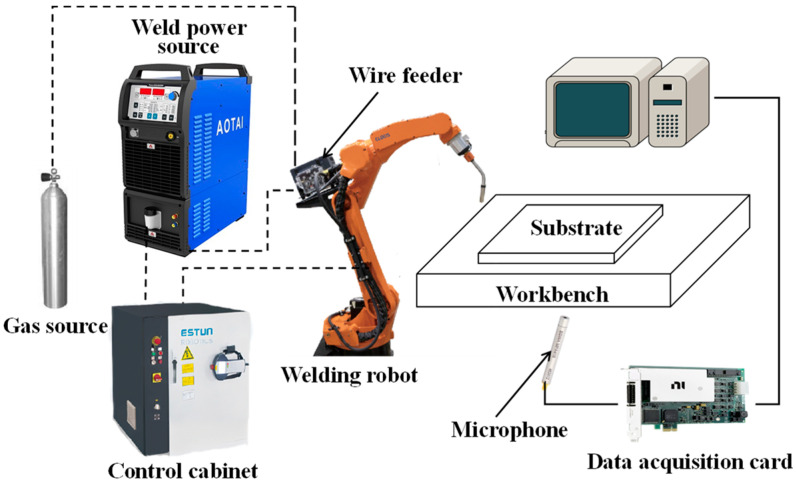
Experimental system.

The experimental setup, illustrated in Figure 2, includes a central computer, an Aotai welding machine (MIG 350 pulse, Aotai Electric Ltd., Jinan, China), a protective gas source, a microphone (MPA416, BSWA Technology Ltd., Beijing, China), an NI data acquisition card (PCIe-6361, National Instruments, Austin, TX, USA), a wire feeder, and a six-axis ESTUN robot (Estun Automation, Nanjing, China). The welding wire used for deposition was H08Mn2SiA with a diameter of 1.2 mm. A single-layer weld bead was deposited on the substrate in the experiment. The experiments were conducted on a Q235 substrate measuring 200 mm × 200 mm × 10 mm. During the welding process, arc acoustic pressure signals were acquired with a sampling rate of 40 kHz by the data acquisition card and microphone. The chemical composition of the welding wire is shown in Table 1. The detailed parameters for the microphone are presented in Table 2. The deep learning model was developed with Intel Core i7-13650HX (2.60 GHz) CPU (Intel, Santa Clara, CA, USA), NVIDIA GeForce RTX4060 (8 GB) GPU (NVIDIA, Santa Clara, CA, USA), Windows11 64bit operating system (Microsoft, Redmond, WA, USA), and python 3.10. Additionally, this study employed a range of libraries, including Pytorch 11.8, NumPy 1.23.5, Pandas 2.0.3, Matplotlib 3.7.1, and others.

### 2.2. Data Acquisition and Pre-Processing

**Figure 3:**
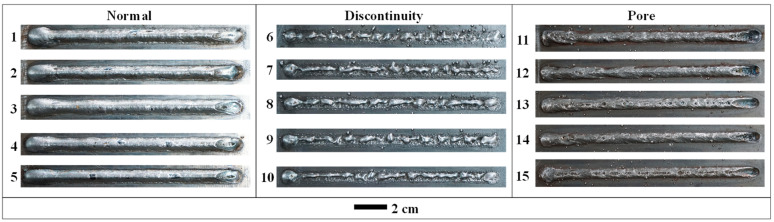
Weld morphology under different process parameters.

Linear heat input (*LHI*) is a parameter that describes the energy absorbed per unit length and plays a crucial role in the overall performance of the formed part. The *LHI* is calculated as follows [31]:

(1) $$LHI=\frac{P}{v}=\frac{UI}{v}$$

where *P* represents the power, *v* denotes the welding speed, *U* indicates the welding voltage, and *I* signifies the welding current. The DED-arc defects identified in this study are discontinuity and pore defects. The quality of the deposited layer can be influenced by inappropriate process parameters, including welding current, welding voltage, welding speed, and protective gas flow [32]. As presented in Table 3, in order to collect both normal and abnormal acoustic signals, different process parameters were used to produce the samples included in the dataset. Through the preliminary experimental exploration, the process parameters of the normal weld bead were obtained. Discontinuity defects are a typical shape defect that compromises the geometrical integrity of the formed parts in DED-arc. Consequently, it is essential to establish real-time monitoring for discontinuity defects. The main reason for discontinuity defects in DED-arc is that the welding speed is too fast, resulting in insufficient molten metal to fill the scanning path. A high welding speed (40–50 mm/s) was employed to gather samples of acoustic signals containing discontinuity defects. A small flow of protective gas increases the intrusion of external gas, resulting in pore defects accompanied by a large number of spattering [33]. Acoustic signal samples with pore defects were collected using a low protective gas flow (0–4 L/min). The macroscopic morphology of the single-layer weld beads is shown in Figure 3. The acoustic signals generated during the DED-arc process exhibited notable differences from the ambient acoustic signals. The acquisition of the acoustic signals commenced when the absolute value of the acoustic pressure exceeded 2.5 Pa and terminated when the absolute value of the acoustic pressure declined below 2.5 Pa.

**Figure 4:**
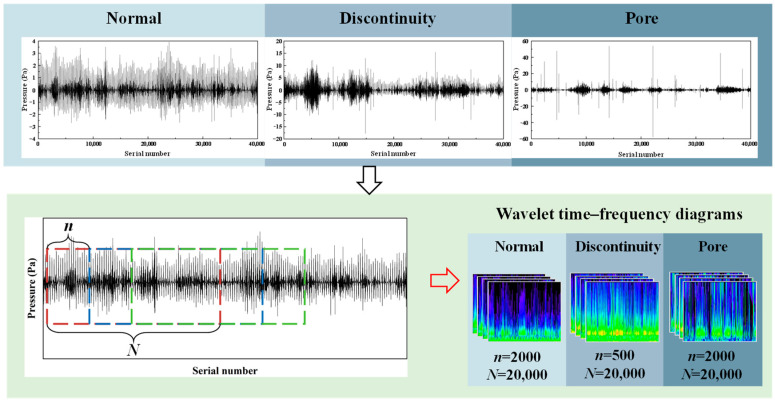
Conversion method of the three types of acoustic signals from 1D signals to 2D time–frequency diagrams.

As illustrated in Figure 4, three categories of acoustic signal samples were collected, namely, normal, discontinuity, and pore. It can be observed that the acoustic signal exhibits distinct characteristics under normal, discontinuity, and pore states. In this study, data enhancement was performed using sliding window sampling, where the width of the sliding window is *N* and the sliding step size is *n*. The 1D acoustic signal was segmented into multiple samples of length *N*. In order to ensure that all samples are of an identical length, it is necessary to pre-calculate the number of samples based on the length of the window, the sliding step, and the length of the acoustic signals. The optimal number of samples is selected to ensure that the window contains a sufficient number of data points, thereby avoiding the issue of insufficient data points in the sliding sampling process. Since discontinuity defects are created by increasing the welding speed, the sampling time for discontinuity defects is much lower than that for the other two categories. To ensure a balanced amount of data for each category in the dataset, when using sliding window sampling for data enhancement operations, the acoustic signals of normal states and pore defects were segmented using a sliding window with a step size of 2000 data points, while the acoustic signals of discontinuity defects were segmented using a sliding window with a step size of 500 data points. This implies that *n* equals 500 for the acoustic signals of normal states and pore defects and 2000 for the acoustic signals of discontinuity defects. In order to ensure that each segmented sample contains the corresponding defective signals, the time of each sample was set to 0.5 s, and the width of the sliding window was set to 20,000 data points, given that the sampling frequency of the acoustic signals was 40 kHz. This implies that *N* is equal to 20,000. One-dimensional acoustic samples extracted from the sliding window were converted into wavelet time–frequency diagrams of 224 × 224-pixel size. This was achieved using the analytic wavelet transform (AWT) with automatic time-step adjustment. AWT is a specific instance of the continuous wavelet transform, employing a complex-valued Morlet wavelet.

Following the application of sliding window sampling and the analytic wavelet transform, a total of 3840 wavelet time–frequency diagrams were generated. Among these, the ratio of normal, discontinuity, and pore datasets was 1:1:1. For each category, the dataset was randomly split into three sections including a training set, a validation set, and a testing set. The distribution of training, validation, and testing sets was 64%, 16%, and 20%, respectively. Table 4 shows the number distribution of training, validation, and testing sets for each category.

### 2.3. CNN Architecture

**Figure 5:**
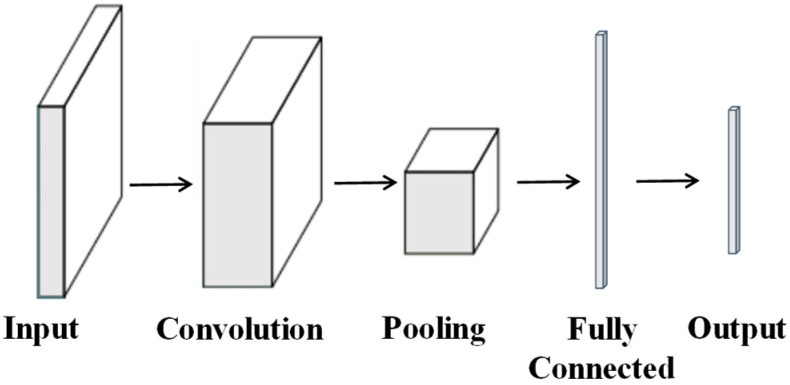
Conventional CNN architecture for classification.

CNNs simulate the visual system of the human brain, automatically extracting image features from the data through convolutional layers [34]. The spatial dimensionality of the data is then reduced through pooling layers, allowing a CNN to learn the features in an image without the need for manual intervention. As research progresses, CNNs are subjected to continual refinement and iteration, resulting in the emergence of a multitude of representative network structures. Currently, CNNs are employed extensively in deep learning for image classification and visual detection. As shown in Figure 5, a conventional CNN architecture for classification comprises a series of successive layers, including an input layer, convolutional layers, pooling layers, fully connected layers, and an output layer [35,36,37]. The input layer is the initial layer of the network and is responsible for receiving raw data. The convolutional layers are responsible for extracting features from the image by using multiple convolutional filters to slide over the input image to produce feature maps. The pooling layers are used to decrease the dimensions of the feature map, leading to a reduction in both parameters and computational workload. The fully connected layers are situated at the end of the network and receive features from the preceding layer, integrating them. The output layer is the last layer in the network and is in charge of producing the final output. When classifying, the number of neurons in the output layer matches the number of target categories, where each neuron corresponds to a category score.

The advent of deep learning has transformed the field of computer vision, with a series of pioneering CNN architectures. In this study, four CNN models including AlexNet, VGG-16, ResNet-18, and MobilenetV3 were employed to achieve the identification of discontinuity and pore defects in DED-arc. MobilenetV3 exhibited the most optimal performance. AlexNet was introduced by Krizhevsky et al. [38], which comprises five convolutional layers and three fully connected layers and uses large convolutional filters. The success of AlexNet has facilitated the widespread use of deep learning in computer vision and has had a profound impact on the design of subsequent deep convolutional neural networks. VGG-16 comprises thirteen convolutional layers and three fully connected layers and uses extremely small convolution filters. Performance was improved by deepening the neural network, which was performed in VGG-16. However, network degradation may occur as the depth of the network increases. He et al. [39] proposed ResNet in 2015, which is an approach that employs a residual learning framework. This framework allows for the construction of deeper networks by introducing shortcut connections. These connections permit signals in the network to propagate directly, bypassing one or more layers, thus resolving the issue of network degradation commonly observed in training deeper networks. This study used ResNet-18, a CNN containing 18 layers of depth. Conventional CNN architectures, which require a significant amount of memory and computational resources, are not suitable for deployment on mobile devices. To address this issue, a lightweight CNN, namely, MobilenetV3, has been proposed [40]. MobileNetV3 introduces depthwise separable convolution, which drastically reduces the number of parameters and processing costs. The convolutional approach comprises two distinct components including a depthwise convolution and a pointwise convolution. The former applies small convolution filters independently to each input channel to extract feature maps, while the latter amalgamates the feature maps based on the outcomes of the depthwise convolution using 1 × 1 filters. In addition, MobileNetV3 employs inverted residuals and a linear bottleneck structure, which further reduces the dimensionality and improves the efficiency of the network. Together with the introduction of the squeeze-and-excitation block, MobileNetV3 enhances feature representation capability without significantly increasing the parameters. This progression of CNN architectures reflects the ongoing quest for models that are not only powerful in terms of representational capacity but also efficient in terms of computational requirements.

### 2.4. Model Hyperparameter Configuration

The learning rate (Lr) is one of the key hyperparameters of deep learning algorithms. The model might not converge at all or converge very slowly if the learning rate is too low. Conversely, if the Lr is set too high, the model might not converge to the least loss value and instead fluctuate around the best answer. The Lr decay strategy was employed in this investigation. The Lr started at 0.001 and was modified every two epochs to become 0.973 times the current value. In order to optimize the model parameters, the AdamW optimizer was employed, which integrates the benefits of momentum and adaptive Lr with improved handling of weight decay. The specifics of the parameters used for training are shown in Table 5. In this study, the parameters for the model training were meticulously calibrated to ensure optimal performance. The training of CNN models was conducted through a series of epochs, where each epoch encompassed a complete pass that the CNN models made over the training set. Within each epoch, the data were divided into batches, and the weights of the CNN models were updated iteratively after processing each batch. The models were configured with a maximum of 300 epochs and a batch size of 32 to optimize learning and generalization on the training set.

## 3. Results and Discussion

### 3.1. Acoustic Signal Analysis

**Figure 6:**
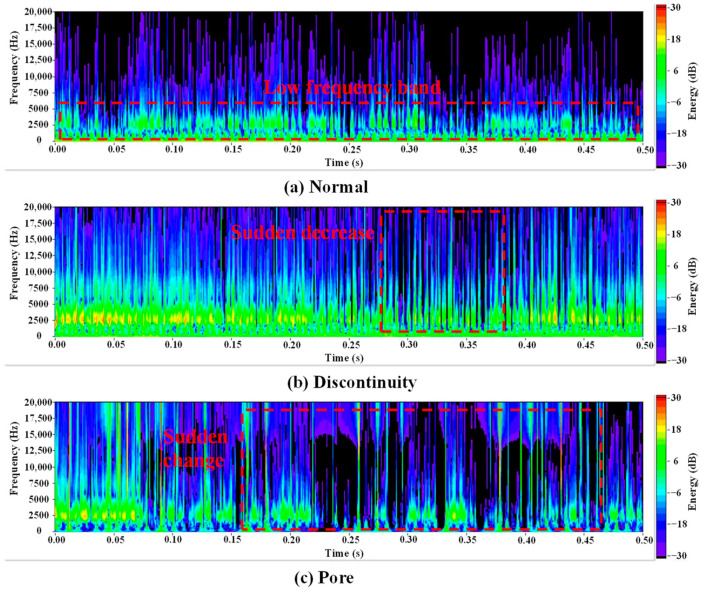
Time–frequency diagrams of acoustic signals. (**a**) Normal, (**b**) discontinuity, and (**c**) pore.

The energy distribution of normal and abnormal acoustic signals has significant differences in both the time and frequency domains. Figure 6 shows typical time–frequency diagrams when the weld bead is in normal, discontinuity, and pore, respectively. As marked in the red dashed line in Figure 6a, the energy of normal acoustic signals is concentrated in the low-frequency band, and the energy changes in the time domain are more stable and regular. As illustrated in Figure 6b, in contrast to the aforementioned normal acoustic signals, acoustic signals of discontinuity have more energy in the high-frequency band and exhibit sudden decreases in energy within the time domain. As can be seen in Figure 6c, the occurrence of a pore introduces high-frequency components to the acoustic signals of pores, resulting in more sudden changes in the time domain. Consequently, the time domain of these signals exhibits greater instability and irregularity.

### 3.2. Evaluation of Model Classification Performance

**Figure 7:**
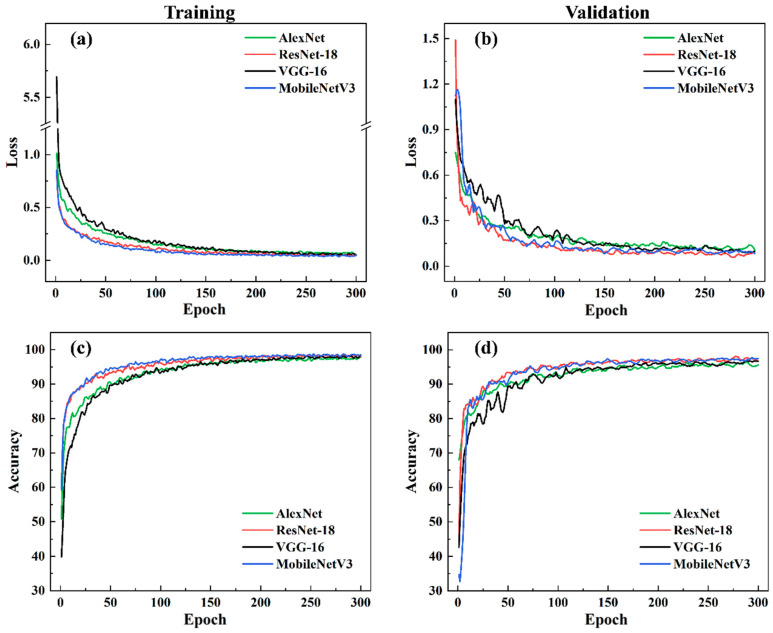
Training curve of CNNs. (**a**) Training loss, (**b**) validation loss, (**c**) training accuracy, and (**d**) validation accuracy.

The value of loss represents a pivotal metric for gauging the difference between the predicted and actual values. Accuracy represents a direct measure of the correctness of the model’s predictions. Loss curves and accuracy curves offer a visual representation of the change in performance during the training of the model. An epoch is defined as a complete cycle in the training process during which the training set is employed to update the weights and biases of the model. Figure 7 shows the variation in loss and accuracy with the number of epochs for the four models in the training and validation sets. It is noticeable that within 150 epochs, there was a significant variation in the loss and accuracy of each model. At around 200 epochs, the training convergence of these models was achieved. All CNN models achieved high accuracy with small loss values. The loss values were within the range of 0.0–0.2, while the accuracy exceeded 95%. In the training phase, the four classes of models were evaluated in comparison to one another. The results indicated that MobileNetv3 and ResNet-18 exhibited quicker convergence rates and superior initial accuracy. VGG-16 exhibited the lowest initial accuracy and the slowest convergence because of its considerable size and numerous model parameters.

**Figure 8:**
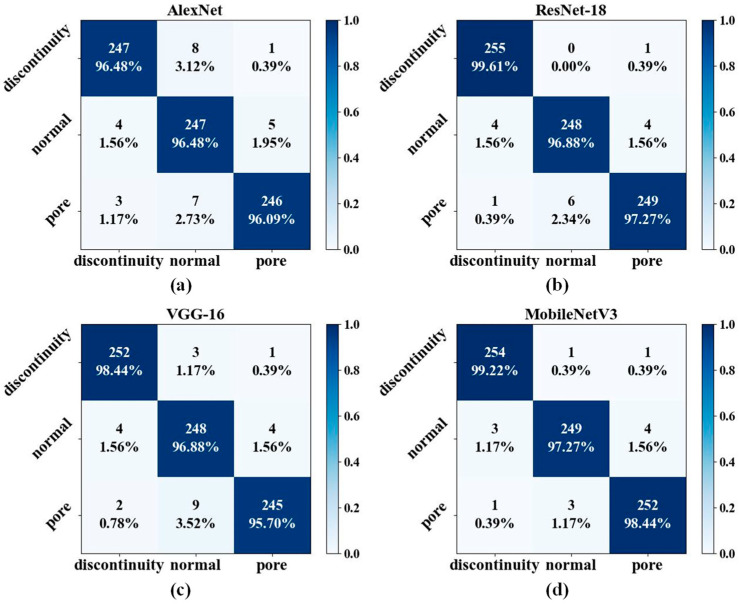
Confusion matrix. (**a**) AlexNet, (**b**) ResNet-18, (**c**) VGG-16, and (**d**) MobileNetV3.

**Figure 9:**
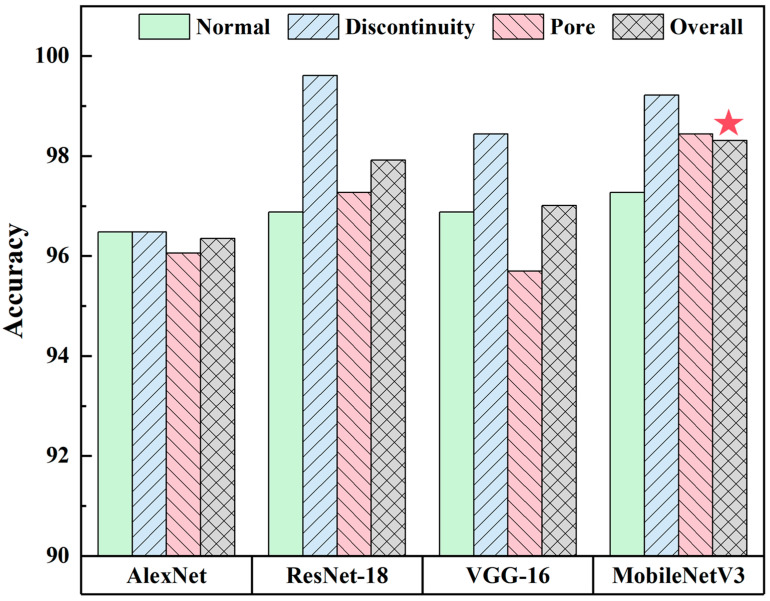
Accuracy of four models for three different categories.

Figure 8 presents the confusion matrix, which offers a more comprehensive understanding of the performance of each model in classification. The predicted categories are represented by the columns in the confusion matrix, and the actual categories are represented by the rows. Figure 9 shows the accuracy of the three categories and the overall accuracy of the model, as indicated by the confusion matrix. As shown by the star symbol in Figure 9, MobileNetV3 achieved the highest overall accuracy of 98.31% for the classification of time–frequency diagrams of acoustic signals. Of all the models in this study, the discontinuous classifications had the highest accuracy. The occurrence of a discontinuity led to an increase in the energy of the acoustic signals, particularly in the high-frequency band, accompanied by a region of sudden decrease in energy in the time domain, resulting in acoustic signals that are significantly different from those of the other two categories. In the case of the normal and pore samples, the occurrence of pore defects is not a constant phenomenon. It is possible to perform sliding-window sampling of pore signals without encountering pore defects in some windows, despite achieving a sliding-window length of 20,000 data points. Consequently, the presence of normal acoustic signals in pore samples represents a further complicating factor, thereby rendering the distinction between the two categories more challenging.

The performance metrics of AlexNet, ResNet-18, VGG-16, and MobileNetV3 are shown in Table 6. *Precision*, *recall*, and the *F*1-*score* are among the performance metrics that can be derived from the confusion matrix and allow for a more thorough evaluation of the classification performance of CNN models. *Precision* is a measurement of a classification model’s accuracy for positive predictions and is calculated as follows:

(2) $$Precision=\frac{TP}{TP+FP}$$

where true positive (*TP*) represents the number of samples that the model correctly predicts as positive categories and false positive (*FP*) represents the number of samples that the model incorrectly predicts as positive categories. *Recall* represents the proportion of positive categories that are correctly predicted by the model across all actual positive categories. The equation for *recall* is presented as follows:

(3) $$Recall=\frac{TP}{TP+FN}$$

where false negative (*FN*) is the number of positive category samples that the model incorrectly predicts as negative categories. The *F*1-*score* is a metric that combines precision and recall and represents the harmonic mean of these two variables. Its calculation formula is as follows:

(4) $$F1-score=2\times \frac{Precision\times Recall}{Precision+Recall}$$

As can be seen from Table 6, all models are able to distinguish among different categories of time–frequency diagrams with a *precision* of over 94%. Regarding *precision*, *recall*, and the *F*1-*score*, AlexNet and VGG-18 demonstrate lower scores, while ResNet-18 and MobilenetV3 exhibit higher scores. Among these models, MobilenetV3 exhibits the most optimal performance, while ResNet-18 is marginally inferior to MobilenetV3.

**Figure 10:**
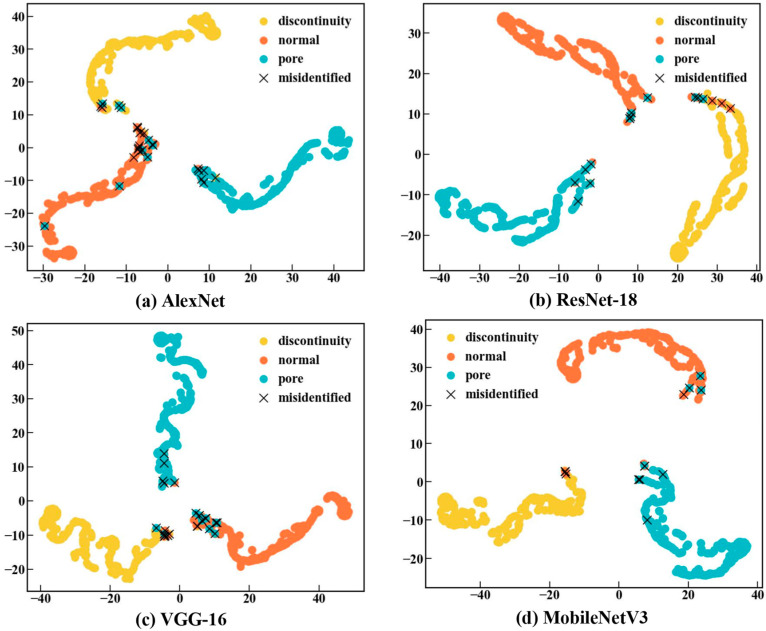
Visualization of CNN models using T-SNE. (**a**) AlexNet, (**b**) ResNet-18, (**c**) VGG-16, and (**d**) MobileNetV3.

In this study, the t-distributed stochastic neighbor-embedding (T-SNE) algorithm was employed to provide a visual representation of the performance of the four classification models. High-dimensional data can be mapped into two-dimensional or three-dimensional space using T-SNE, thereby enabling the visualization of high-dimensional features [41]. Figure 10 shows the feature distribution of the output results of each model following dimensionality reduction via T-SNE. It can be clearly seen that the output features of each category of each model are clustered and that each category is separated. This indicates that the CNN models are capable of effectively extracting features from the time–frequency diagrams and of effectively differentiating among the different categories based on the learned features. Furthermore, the degree of separation of the categories in these four models provides a visual demonstration of the classification performance of each model. Figure 10 illustrates that Mobilenetv3 exhibits the highest category separation accuracy and the least false identifications, indicating that it has optimal performance.

In addition to the aforementioned metrics, the number of parameters, training time, and detection time are also important for assessing the performance and efficiency of the models. In the field of deep learning, the differing structures and parametric quantities of various CNN models significantly affect a model’s complexity, accuracy, training efficiency, and inference speed. The three evaluation metrics mentioned above are presented in Table 7. AlexNet and VGG-16 are both older architectures with a considerable number of model parameters, particularly VGG-16, which has 134.28 million parameters. This is due to the fact that VGG-16 has a deeper network structure, comprising thirteen convolutional layers and three fully connected layers. As the depth of the network increases, the number of convolutional filters grows rapidly, which results in a large number of parameters. The training time and detection time of VGG-16 are considerably longer than those of other models because of the large number of parameters, deep structure, inefficient gradient descent, and large memory footprint. AlexNet uses a larger convolutional filter (11 × 11) for its first convolutional layer and contains three fully connected layers, resulting in 57.02 million parameters. A deeper network structure means that more layers are subject to forward and backpropagation, which increases the computational burden during training and detection. Despite the considerable number of parameters, AlexNet has a short training time and detection time because of its shallow network structure. The ResNet-18 and MobileNetV3 models have a reduced number of parameters, with the latter having a parameter count of only 1.52 million. ResNet-18 introduces shortcut connections and has only one fully connected layer. These measures reduce the number of parameters while improving the accuracy of the model. MobileNetV3 has the fewest parameters and the shortest training time, primarily because of its innovative architectural design, which significantly reduces the number of parameters and computational complexity, thus shortening the training time and accelerating the inference of the model. It should be noted that the training time of the model is also contingent on the performance of the hardware. The utilization of an advanced GPU can result in a reduction in the training time of CNN.

In consideration of each performance metric, MobileNetV3 demonstrated the most optimal performance among the four models studied for the task of acoustic signal-based defect identification in DED-arc. This model achieved the highest classification accuracy, the fewest parameters, the shortest training time, and an extremely fast detection rate.

## 4. Conclusions

This study proposed a novel method for identifying defects in DED-arc based on acoustic signals, employing wavelet time–frequency diagrams. Furthermore, the energy distribution of acoustic signals in different states was analyzed based on wavelet time–frequency diagrams. In order to diagnose discontinuity and pore defects in DED-arc, an in situ monitoring system based on acoustic signals utilizing a CNN was developed, and the defects were successfully identified. The proposed method, which provides robust defect identification tools, facilitates the accelerated adoption of DED-arc across a range of industries, particularly those with exacting quality and performance requirements. This method enhances the stability and reliability of the manufacturing process and propels DED-arc to a higher level of automation and intelligence. The following paragraphs present a summary of the key findings.

1. The analysis of acoustic signals revealed that the energy distribution of normal and abnormal acoustic signals is significantly different in both the time and frequency domains. The energy distribution of normal acoustic signals is more stable. The acoustic signals of discontinuity defects have more energy in the high-frequency band compared with normal acoustic signals. Additionally, there is a region of sudden decline in energy within the time domain. The acoustic signals of pore defects are characterized by high levels of instability and irregularity.
2. Four different CNN architectures were compared, namely, AlexNet, VGG-16, ResNet-18, and MobileNetV3, to identify the most effective model for the classification task in this study. The four CNN models were trained on a dataset consisting of time–frequency diagrams. MobileNetV3 achieved a classification accuracy of 98.31%, while AlexNet, ResNet-18, and VGG-16 achieved 96.35%, 97.92%, and 97.01%, respectively. The results demonstrate that the methodology proposed in this study is an effective means of identifying defects in the DED-arc process.
3. In terms of accuracy, number of model parameters, training time, and detection time, the MobilenetV3 model achieved the best performance. It had the highest classification accuracy, the smallest number of parameters, the shortest training time, and a fast detection rate.

In summary, the defect identification method of DED-arc based on acoustic signals facilitates the automation of production and an enhancement in production quality in DED-arc. In future research, the identification accuracy will be further improved by data fusion of visual and acoustic signals. This method can be extended to other areas of metal additive manufacturing, such as laser powder bed fusion, laser-directed energy deposition, and electron beam free-form manufacturing.

## Figures and Tables

**Table 1:** H08Mn2SiA chemical composition (wt.%).

Alloy	C	Mn	Si	S	P	Cr	Ni	Cu	Fe
H08Mn2SiA	0.08	1.97	0.72	0.02	0.02	0.04	0.01	0.2	Bal

**Table 2:** Parameters of the MPA416 microphone.

Frequency Response	Dynamic Range	Sensitivity	Output Interface
20 Hz–20 kHz	29 dB–127 dB	48.3 mV/Pa	SMB

**Table 3:** Process parameters for acquiring normal and abnormal acoustic signals.

Status	Welding Current (A)	Welding Voltage (V)	Welding Speed (mm/s)	LHI (J/mm)	Protective Gas Flow (L/min)
Normal	180	30	8	675	20
	180	28	8	630	20
	180	26	8	585	20
	166	26	8	540	20
	152	26	8	495	20
Discontinuity	220	30	40	165	20
	228	29	40	165	20
	236	28	40	165	20
	244	27	45	165	20
	254	26	50	165	20
Pore	180	30	8	675	4
	180	28	8	630	4
	180	26	8	585	2
	166	26	8	540	2
	152	26	8	495	0

**Table 4:** Data for training, validation, and testing.

Category	Training Set	Validation Set	Testing Set
Normal	820	204	256
Discontinuity	820	204	256
Pore	820	204	256

**Table 5:** Training parameters of the CNN.

Parameters		Value
Max epochs		300
Batch size		32
Initial leaning rate		0.001
Learning rate decay strategy		Lr decay for every two epochs: Lr × 0.973
Optimizer (AdamW)	Weight decay	0.001
	Epsilon	1 × 10^−8^
	Betas	(0.9, 0.999)

**Table 6:** *Precision*, *recall*, and the *F*1-*score* of different deep neural networks for wavelet time–frequency diagrams of acoustic signals (%).

Model	Category	*Precision*	*Recall*	*F*1-*Score*
AlexNet	Discontinuity	97.24	96.48	96.86
	Normal	94.27	96.48	95.37
	Pore	97.62	96.09	96.85
ResNet-18	Discontinuity	98.08	99.61	98.84
	Normal	97.64	96.88	97.25
	Pore	98.03	97.27	97.65
VGG-16	Discontinuity	97.67	98.44	98.05
	Normal	95.38	96.88	96.12
	Pore	98.00	95.70	96.84
MobileNetV3	Discontinuity	98.45	99.22	98.83
	Normal	98.42	97.27	97.84
	Pore	98.05	98.44	98.25

**Table 7:** Other metrics to assess model performance.

Model	Parameters (M)	Average Training Time per Epoch (s)	Detection Time per Image (s)	Image Input Size (px^2^)
AlexNet	57.02	16.61	0.0111	224 × 224
ResNet-18	11.18	17.63	0.0148	224 × 224
VGG-16	134.28	68.94	0.0667	224 × 224
MobileNetV3	1.52	13.01	0.0217	224 × 224

## Data Availability

All data required to reproduce the results can be obtained from the corresponding author upon a reasonable request.
